# HOSPITAL CARE USE IN OLDER ADULTS: THE ROLE OF PSYCHOLOGICAL AND SOCIAL FACTORS

**DOI:** 10.1093/geroni/igz038.2709

**Published:** 2019-11-08

**Authors:** Viviane Straatmann, Serhiy Dekhtyar, Bettina Meinow, Laura Fratiglioni, Amaia Calderon-Larranaga

**Affiliations:** 1 Aging Research Center - Karolinska Institute, Stockholm, Sweden, Sweden; 2 Aging Research Center, Department of Neurobiology, Care Sciences and Society, Karolinska Institutet and Stockholm University, Stockholm, Sweden

## Abstract

Although older people’s health status is the main determinant of healthcare use, there has been little research on how psychosocial factors relate to healthcare utilization. We explored the extent to which psychological and social aspects predict the use of hospital care in an older Swedish population. 2867 people ≥60 years from the Swedish National study on Aging and Care in Kungsholmen (SNAC-K) were followed from baseline (2001-2004) for four years. We created standardized indexes of psychological well-being, and social well-being. Binomial negative mixed models were used to estimate the association of psychological and social indexes with hospital care use (i.e. unplanned hospital admissions [UHA], 30-day readmissions [30DR] and length of stay [LOS]). Individuals with a psychological well-being score above the median had less UHA (IRR 0.43, 95%CI 0.20-0.93) and lower LOS (IRR 0.18, 95% 0.06-0.58), even after full adjustment. High levels of social well-being were also protective for UHA and LOS in the minimally adjusted model, but not after adjusting by life style and personally traits. Relative to individuals with poor well-being on both indexes, those with rich psychological and poor social well-being had reduced hospital care use (IRR 0.44 95%CI 0.24-0.84; IRR 0.23, 95%CI 0.08-0.67, respectively), and even further in those with rich psychological and social well-being (IRR 0.33 95%CI 0.14-0.75; IRR 0.10, 95% 0.02-0.45, respectively). No statistically significant association was found with 30DR. Provided the importance of psychosocial aspects in predicting UHA and LOS, targeting the former could be a strategy for reducing healthcare use and, eventually, costs.

